# Use of Idarucizumab to Revert the Anticoagulant Effect of Dabigatran in Heart Transplant Surgery: An Institutional Experience

**DOI:** 10.1155/2020/6927423

**Published:** 2020-03-06

**Authors:** Álvaro Herrera-Escandón, Orlando Castaño-Cifuentes, Carlos A. Plata-Mosquera

**Affiliations:** ^1^Internal Medicine Department, Universidad del Valle, Cali, Colombia; ^2^Cardiology Department, DIME Clínica Neurocardiovascular, Cali, Colombia; ^3^Heart Failure and Cardiac Transplant Unit, DIME Clínica Neurocardiovascular, Cali, Colombia

## Abstract

Heart transplant is a surgical procedure with a high risk of perioperative bleeding in patients with a previous history of sternotomy, congestive liver disease, and/or use of oral anticoagulants. Anticoagulation is usually done with coumarin agents (warfarin, acenocoumarol), while on the waiting list, vitamin K is available allowing for partial reversal of the anticoagulant effect, although with variable INR and risk of uncontrolled bleeding. Direct oral anticoagulants have emerged as an alternative to the use of coumarins in patients with nonvalvular atrial fibrillation (NVAF). The main disadvantage of this group of drugs is that there was no specific reversal agent available that would allow an urgent reversal of the anticoagulant effect. The recent commercialization of idarucizumab (specific reversal agent) has allowed patients with NVAF on the waiting list for heart transplant to be treated with dabigatran. We present the case of a patient with advanced chronic heart failure and NVAF anticoagulated with dabigatran, who underwent urgent heart transplant after administration of idarucizumab, without complications derived from its use or from anticoagulation.

## 1. Introduction

New oral anticoagulants have emerged as an alternative to warfarin in the treatment of venous thromboembolic disease and as prevention of thromboembolic events in nonvalvular atrial fibrillation (NVAF). Dabigatran inhibits coagulation-activated factor II while apixaban and rivaroxaban inhibit factor Xa [1]. These anticoagulants are classified as direct-acting anticoagulants and are as effective as warfarin in ischemic event prevention with a better security profile [2, 3]; however, there was no available antidote that allowed for urgent reversal of the anticoagulant effect to realize urgent invasive procedures, mainly surgery.

Idarucizumab is a reversal agent specific for dabigatran. It is a fragment of immunized monoclonal antibody (Fab) that attaches to dabigatran and its metabolites with high affinity. It is approximately 350 times more potent than the affinity of dabigatran for thrombin. Compounded idarucizumab-dabigatran is characterized by a fast association constant and an extremely slow dissociation constant, giving way to a very stable compound. Idarucizumab attaches to dabigatran and its metabolites in a potent and specific manner, neutralizing its anticoagulant effect. It is indicated in adult patients treated with dabigatran etexilate when a fast reversal of its anticoagulant effects is needed (emergency surgical interventions or in the case of potentially deadly or uncontrolled hemorrhaging) with good clinical results and equal bleeding rates as patients not anticoagulated.

Patients with chronic heart failure (CHF) present with atrial fibrillation rhythm in as much as 30% of cases [4, 5]. The benefits offered by NOAC by not requiring monitorization of therapeutic effects, and having less variability between subjects and having a more predictable effect compared to warfarin make them an option in NVAF treatment.

We present a case of a patient with advanced chronic heart failure and NVAF anticoagulated with dabigatran who was subjected to an emergency heart transplant after idarucizumab administration.

## 2. Clinical Case Description

A 60-year-old, group A Rh-negative male had been waitlisted for heart transplant due to dilated cardiomyopathy of nonischemic origin, stage D with INTERMACS 5 profile with an ejection fraction of 20%.

Disease debut in the year 2012 with embolic ischemic cerebrovascular event with intracavitary apical thrombus causes the event, with small sequela in left upper extremity (monoparesis). Patient received medical treatment in accordance to clinical practice guidelines, including resynchronization therapy (150 msec QRS) and defibrillation for primary prevention. The patient had advanced heart failure, with NYHA IIIA class of symptoms and VO2 peak less than 10 ml/kg/min in cardiopulmonary exercise test.

Evolution of the latest years shows multiple hospitalizations due to congestion, hypoperfusion, renal dysfunction, and arrhythmic storm with discharges appropriate to defibrillator treated with amiodarone.

Of note in the first follow-up is the development of atrial fibrillation with ischemic risk calculated CHADS2 VASC of 4 (heart failure, AHT, ischemic stroke) and risk of bleeding HASBLED of 1 in anticoagulation treatment with dabigatran 150 mg every 12 hours since 2015. We did not use any type of heparin prior to the procedure.

After 6 months on the waiting list, an effective and compatible donation alert was activated at 22:00 hours. Patient takes the last dose of dabigatran at 09:00 hours (13 hours) and administration of reversal agent determined previous to heart transplant surgery.

Upon arrival, the first laboratory exams are taken noting a creatinine elevation compared to basal state as single additional finding.

Reversal with bolus of total dose of 5.0 g from 2 vials (2.5 g) of idarucizumab at 23:57 and 00:15, respectively, was passed in the surgical room.

After 3:46 hours of ischemic time of the graft, with subjectively considered normal bleeding by the surgical team, the patient is transferred to the intensive care unit (ICU). We had standard bleeding during perioperative state, and the patient needed 3 more units of erythrocyte concentrate after immediate surgery, without additional transfusion requirements after 24 hours in ICU. Our group uses intraoperative hemostatic factors as a surgical adhesive, careful hemostasis prior to sternal closure.

Patient is required with inotropic infusion in accordance with transplant protocol with 0.03 mcg/kg/min of isoproterenol and 7.5 mcg/kg/min of dobutamine. Immunosuppression induction performed with 20 mg of basiliximab on days 0 and 4 and extubation was programmed upon 24 hours of surgical procedure. At 48 hours of ICU arrival, mediastinal drains were removed with a total bleed of 295 ml in the first 6 hours, 835 ml at 30 hours, and finally 1045 ml at 48 hours, thereby completing security protocol for retrieval. Coagulation times, platelet count, hemoglobin, and creatinine behavior were adequate ([Supplementary-material supplementary-material-1]).

The total ICU stay time was 7 days and protocol prophylaxis was started with valganciclovir, trimethoprim sulfamethoxazole, and nistatin.

On the tenth day, herpes zoster is evidenced and successfully treated on T8 dermatome; this is a single finding during hospital stay.

The 15^th^ day endomyocardial biopsy was negative for cellular and humoral rejection, with adequate biventricular function of graft. Patient was released 17 days after transplant and until now lives free of further clinical findings. We used the usual thromboprophylaxis with enoxaparin during the hospital stay.

## 3. Discussion

The idarucizumab effectively reversed the anticoagulant effect of dabigatran in patient taken to heart transplant, obtaining good hemostasis during surgery and without presenting bleeding requiring transfusion support post operation. Normal coagulation tests and an adequately functioning coagulation system are key to contributing to a favorable outcome in patient subject to mayor surgery.

The dabigatran reaches its peak plasma anticoagulant activity upon 2 hours of ingestion, having a bioavailability of between 3 and 7% and attaching 35% to plasma proteins, with up to 80% of renal elimination, with a half-life of between 12 and 17 hours if renal function is normal [6]. Different control studies and meta-analyses have demonstrated its efficacy as an anticoagulant and its security profile [1]. Current international guides recommend it as thromboembolic prevention in patients with atrial fibrillation [7]. Until recent years, the main limitation in the use of these medicines was the lack of a reversal agent and the inability to monitor its anticoagulant effect through conventional exams measuring coagulation and those that approximate the measurement of its activity are not available in our region.

As opposed to warfarin, the lack of an effective reversal agent in restoring hemostasis in the case of severe hemorrhaging or emergency or urgent surgery was seen for many years as a limitation in the use of dabigatran and other direct anticoagulants. The arrival of idarucizumab supported in RE-VERSE AD [8] study opened the field for those patients with mayor hemorrhage who require urgent surgery to establish hemostasis within minutes of its administration.

Our case is the first reported in Colombia and in Latin America on the use of idarucizumab in heart transplant. Two similar cases to ours were found in the literature [9, 10] in which the successful use in patients with mayor surgery is documented; however, although in the REVERSE-AD study, there were no patients with heart transplant; these 3 reported cases in the medical literature suggest that this medication is equally as effective and safe in this population [11], which has high prevalence of atrial fibrillation [12].

In conclusion, idarucizumab is an effective and safe specific reversal agent in patients anticoagulated with dabigatran including those who have been subjected to mayor surgery such as heart transplant. The fact of having a reversal agent makes dabigatran a medication of choice in these patients with advanced heart failure who require anticoagulation and need emergency surgery.

## Supplementary Materials

Supplementary Materials Creatinine behavior, coagulation times, hemoglobin, and platelets compared to thoracic tube production after cardiac transplant.
