# Supplementary material for: Use of Idarucizumab to Revert the Anticoagulant Effect of Dabigatran in Heart Transplant Surgery: An Institutional Experience
Source: Case Rep Cardiol. 2020 Mar 6;2020:6927423. doi: 10.1155/2020/6927423 (PMC7081017; doi:10.1155/2020/6927423)
Supplement: Supplementary Materials — Creatinine behavior, coagulation times, hemoglobin, and platelets compared to thoracic tube production after cardiac transplant. [file 6927423.f1.docx]

**Supplement 1**

| **Day Post-operation** | **Tube production day (CC)** | **Creatinine (mg/dl)** | **PT (sec)** | **INR** | **PTT (sec)** | **Hemoglobin (Gr/dl)** | **Platelets** |
| --- | --- | --- | --- | --- | --- | --- | --- |
| 1 | 295 | 1.5 | 13 | 1.1 | 30 | 8.1 | 122000 |
| 2 | 695 | 1.1 | 12 | 1 | 25 | 8.8 | 108000 |
| 3 | 240 | 1 | 12 | 1 | 24 | 8.6 | 113000 |
| Egreso | - | 1.1 | - | - | - | 10.9 | 238.000 |

***Table 1:*** *Creatinine behavior, coagulation times, hemoglobin and platelets compared to thoracic tube production after cardiac transplant*
